# Beneficial Bacteria Isolated from Grapevine Inner Tissues Shape *Arabidopsis thaliana* Roots

**DOI:** 10.1371/journal.pone.0140252

**Published:** 2015-10-16

**Authors:** Enrico Baldan, Sebastiano Nigris, Chiara Romualdi, Stefano D’Alessandro, Anna Clocchiatti, Michela Zottini, Piergiorgio Stevanato, Andrea Squartini, Barbara Baldan

**Affiliations:** 1 Dipartimento di Biologia, Universita degli Studi di Padova, Padova, Italy; 2 Dipartimento DAFNAE - Department of Agronomy Food Natural Resources Animals and Environment, Legnaro (PD), Italy; Centre National de la Recherche, FRANCE

## Abstract

We investigated the potential plant growth-promoting traits of 377 culturable endophytic bacteria, isolated from *Vitis vinifera* cv. Glera, as good biofertilizer candidates in vineyard management. Endophyte ability in promoting plant growth was assessed *in vitro* by testing ammonia production, phosphate solubilization, indole-3-acetic acid (IAA) and IAA-like molecule biosynthesis, siderophore and lytic enzyme secretion. Many of the isolates were able to mobilize phosphate (33%), release ammonium (39%), secrete siderophores (38%) and a limited part of them synthetized IAA and IAA-like molecules (5%). Effects of each of the 377 grapevine beneficial bacteria on *Arabidopsis thaliana* root development were also analyzed to discern plant growth-promoting abilities (PGP) of the different strains, that often exhibit more than one PGP trait. A supervised model-based clustering analysis highlighted six different classes of PGP effects on root architecture. *A*. *thaliana* DR5::GUS plantlets, inoculated with IAA-producing endophytes, resulted in altered root growth and enhanced auxin response. Overall, the results indicate that the Glera PGP endospheric culturable microbiome could contribute, by structural root changes, to obtain water and nutrients increasing plant adaptation and survival. From the complete cultivable collection, twelve promising endophytes mainly belonging to the *Bacillus* but also to *Micrococcus* and *Pantoea* genera, were selected for further investigations in the grapevine host plants towards future application in sustainable management of vineyards.

## Introduction

Endophytes are conventionally defined as bacteria or fungi that live inside a plant without causing any negative effects to their host [1–5]. They are isolated upon disinfection of the plant surface and, collectively, constitute the ensemble of the microbial genomes that are found inside the various organs and tissues of a plant [6].

These micro-organisms receive from the plant nutrients and a protected environment where to grow and establish larger populations; in turn, they produce factors that facilitate the growth of the host as well as its resistance to pathogen infections. The plant growth-promoting effect of endophytic bacteria, that live inside most, if not all, plant species, occurs through the concerted activity of phytostimulation, biofertilization and biocontrol [7]. Phytostimulation is the gain in plant growth that is obtained either through the modulation of the levels of endogenous hormones of the host plant, or through the direct expression of phyto-hormones produced by endophytes [7, 8]. Many plant-associated bacteria can indeed synthesize gibberellins, cytokinins, auxins, ethylene and/or its precursor 1-aminocyclopropane-1-carboxylate (ACC) (for a review see [9]). Among these phyto-hormones, auxins and, in particular, indole-3-acetic acid (IAA) coordinate various developmental processes in the plant, including that of modifying roots morphology. In this organ, the ensuing extension of its exchanging surface has a major impact on the ability of the plant to acquire nutrients and water, which in turn also impacts on the development of the organs of the plant that grow above ground. It is now well-established that IAA biosynthesis is a widespread trait among bacteria of different taxa associated with plants which, in this way, exert a pivotal role in growth and development of their host [10–13]. More specifically, several studies have attested the ability of plant growth-promoting bacteria to change the length of the primary and lateral roots, as well the number of root hairs, which, altogether, increase the root surface of the plant [14, 15]. Biofertilization is the gain in plant growth that is obtained through the increased availability of nutrients [16]. Plant growth-promoting endophytes (PGPEs) can solubilize phosphorus by releasing low molecular weight acids that can chelate the metal cation of phosphorus salts, thereby increasing the bio-availability of this essential element to plant tissues [17]. In addition, phosphorus transformation is obtained through the expression of phytase and phosphatase enzymes, which yield phosphate ions from organic compounds, such as phytic acid [9, 18]. PGPEs are also a source of ammonium; this key nutrient is produced by transforming organic or inorganic nitrogen-containing molecules, which is then assimilated into aminoacids and other biomolecules; moreover this PGP trait can be involved in biocontrol mechanisms as well [8, 9, 19].

Another positive effect that bacterial endophytes have on plant growth is exerted through the production of siderophores; these are small, high-affinity metal-chelating compounds that, once secreted, bind insoluble iron ions to form siderophore-Fe complexes that are taken up either by the bacteria or by the plant. Siderophores lead to an effective increase in the amount of bio-available iron to the inner tissues of the plant; further they provide a nutritional competitive advantage against pathogens by establishing a biocontrol activity that, albeit indirect, is advantageous for the health and growth of the host [8, 20, 21]. The secretion of lytic enzymes, such as cell wall degrading enzymes (endoglucanase), is also considered to have a bio-controlling effect; these enzymes are indeed effective against the cell wall of many pathogenic microorganisms and, by loosening the plant cell walls and contacts among plant cells, facilitate the entry and the spread within the various plant tissues [22]. PGPEs have, therefore, a considerable potential in agriculture as a complement to chemical fertilizers and pesticides. As such, understanding how PGPEs promote plant development and physiology is imperative to translate basic research in the development of novel sustainable agricultural systems [23]. Within this context, optimizing the use of PGPEs in the cultivation of *V*. *vinifera* might contribute to make the cultivation of grapevine more sustainable, less dependent on chemical fertilizers, and improve the management of vineyards.

To this goal, we set out to study the bacterial endophytes that colonize the Prosecco grapevine, cv Glera, that is grown in the Italian region of Veneto. More specifically, in collaboration with the “Consorzio per la Tutela del Conegliano-Valdobbiadene DOCG”, we characterized the entire culturable collection of endophytes found in *V*. *vinifera* cv. Glera, which is composed by 377 bacterial strains, by testing the ability of each strain to solubilize phosphate, produce ammonia, secrete siderophores and lytic enzymes and synthesize a key phytohormone, IAA. In addition we evaluated their effects on root development in the model plant *A*. *thaliana*. Altogether, the data reported in this study show that a comprehensive biochemical and functional approach can be used to identify the most valuable endophytic bacterial strains that may potentially be useful in Prosecco vineyards as biofertilizer and biocontrol agents. Their future utilization is likely to yield innovative environmentally-friendly applications in this highly valued crop.

## Materials and Methods

### Strains

377 endophytic strains, isolated from *V*. *vinifera* tissues [24] and stored in the laboratory were biochemically characterized and tested for plant growth promotion activity. Bacteria were grown in Nutrient Broth agar plates shortly before their use. All described experiments were performed twice with three replicates.

### Cellulose degradation assay

Secretion of enzymes for cellulose degradation was investigated using an in vitro assay [22, 25]. 2 μL of bacterial colonies, re-suspended in 1 mL of 10 mM MgSO_4_, were placed in plates containing solid nutrient medium PCA supplemented with 0.25% of carboxy-methyl cellulose (CMC). Plates were incubated at room temperature for 72 hours and after extensively washed to clean the medium surface. Plates were stained with 1 mg/mL Congo Red solution for 30 minutes. To improve contrast, several washings with a de-staining solution (1 M NaCl) were performed for about 30 minutes. Formation of a clear zone in correspondence of colonies indicated CMC degradation.

### Evaluation of ammonia production

Fresh cultures were inoculated in 10 mL peptone water (10 g/L peptone, 5 g/L sodium chloride) and incubated for 48-72h at 30°C. A volume of 0.5 mL of Nessler’s reagent was added to each tube. Change from yellow to brown was considered as a positive result while no change in colour was scored as negative [25].

### Phosphate solubilizing ability

Phosphate solubilization was assayed following the method of Goldstein [26]. Bacterial isolates were inoculated onto plates with sterilized Pikovskaya’s medium containing tri-calcium phosphate and incubated at 30°C for 72h. Formation of a clear zone around the colony indicated phosphate solubilization activity [27].

### Siderophore production assay

Siderophore production was qualitatively determined by CAS agar assay [28]. Bacteria (a 5μL amount of an overnight culture) were streaked on CAS agar medium and incubated for 72 h in the dark at 30°C. Secretion of siderophores by bacteria was visualized by a colour change of the medium from blue to orange/yellow as the produced siderophores, binding iron more tightly than the ferric complex of Chrome Azurol S, removed iron from the CAS agar medium.

### IAA production

Production of IAA was tested using a colorimetric method described by Patten and Glick [29] with some modifications; 5 mL of bacterial suspension in NB medium amended with 5 mM tryptophan, 0.06% SDS and 1% glycerol [30] were incubated for 72h, centrifuged at 12,000 rpm for 5 min to pellet bacteria and obtain the supernatant. 2 mL of FeCl_3_-HClO_4_ were added to 1 mL of the supernatant. After 25 minutes at room temperature, absorbance at 530 nm was measured. The concentration of IAA from each culture medium was calculated from a pure IAA standard curve.

### *Arabidopsis* root growth

Endophyte plant growth-promoting traits were assayed *in vitro* on the *A*. *thaliana* ecotype Columbia (Col-0) model plant: 13 seeds/squared plate were planted in ½MS medium without sugar containing (for inclusion) one of the 377 strains each to a concentration of 10^6^ cell/mL of medium. Non-inoculated seeds served as a negative control and 50 nM IAA-treated seeds were also used to compare the endophyte-induced effects on root architecture. This experiment was conducted twice with three replicates. Two weeks after seeding, plates were scanned with the Epson Perfection V700 flatbet Photo Scanner (Digital ICE Technologies) and 300 dpi TIFF 24-bit color images were acquired. Root development was analyzed by means of the WinRhizo^®^ software (Regent Instruments Inc., Quebec Canada) taking in account length, area and diameter of the roots. At this final time point of growing (two weeks), plant roots did not interact or touch each other as it is possible to see from representative images of the plates in S1 Fig. This provide evidences that the measured parameters were not affected by the number of seedlings per plate. At this moment, for each treatment condition, the total fresh weight of plantlets was also recorded.

### β-Glucuronidase histochemical assay

The DR5::GUS reporter plants [31] were grown in presence of the IAA producer strains as previously described. Plants were thus analyzed for β-Glucuronidase (GUS) activity by histochemical staining. Samples were incubated in the reaction medium (2 mM X-Gluc, 0.05% Triton X-100, 5 mM K_3_Fe(CN)_6_, 5 mM K_4_Fe(CN)_6_ x 3H_2_O, 10 mM EDTA, 50 mM sodium phosphate buffer pH 7.0) for 16h at 37°C. Seedlings were mounted in a Chloral hydrate solution and pictures were captured with a Leica 5000B microscope using Normarsky correction (DIC).

### 4MU quantitative fluorimetric assay

The *Arabidopsis* DR5::GUS reporter plantlets [31], grown as previously described, were analyzed for β-Glucuronidase activity by 4-Methylumbelliferone (4MU) fluorimetric assay, performed at different developmental stages. 20 seedlings per treatment were pestled and total protein content was incubated several times in the reaction medium (2 mM 4-MU-glucuronide, 0.05% Triton X-100, 10 mM EDTA, 50 mM sodium phosphate buffer pH 7.0) at 37°C. 4MU fluorescence was measured by LS-55 Luminescence Spectrometer (Perkin-Elmer) with an excitation wavelength of 365 nm and an emission wavelength of 455 nm, using a slit width of 5 nm. Experiments were performed at least in triplicate and each sample consisted of 20 seedlings.

### Statistical methods

We used a model based cluster algorithm [32] to appropriately define the number of groups according to the area, diameter and length of the root morphology. The method is based on the estimation of different models (mixed Gaussian distribution) characterized by different numbers of groups; thus, the best model (the best number of groups) is selected according to the Bayesian Information Criterion or BIC [33]. This analysis was performed with R software (http://www.r-project.com) with the mclust package [32, 33].

To test the differences between the means for each variable (area, length and diameter of the root morphology) among the groups we used a t-test with Tukey multiple testing correction [34].

## Results

### Endophytes isolated from Glera possess direct and indirect PGP activities

A total of 377 bacterial strains, isolated from inner tissues of grapevine Glera plants [24], were tested for some PGP abilities, choosing among those activities that, also in grapevine, could directly facilitate plant nutrient acquisition and modulate plant hormone IAA levels or indirectly act as biocontrol against pathogens The distribution of the considered activities among the identified Classes [24] is shown in Table 1 and an overview of chosen PGP traits is given for all 377 strains in S1 Table. 33% (124 strains out of 377) of all assayed endophytes mobilized phosphate from the substrates and 39% (147 strains out of 377) produced ammonia when cultured in peptone water. By using CMC as analogue of plant cellulose, 22% (82 out of 377) secreted enzymes with endoglucanase activity and the CAS agar test demonstrated that 38% (144 out of 377) of the strains secreted siderophores. 17 strains out of 377, investigated by Salkowsky’s assay, synthetized IAA and IAA-like molecules in a concentration ranging from 1.1 to 100.7 μg/mL, when grown in presence of L-tryptophan 5 mM (S1 Table).

**Table 1:** PGP traits of isolated bacteria across different Classes.

		Classes					
PGP traits		*Actinobacteria*	*α-proteobacteria*	*Bacilli*	*β-proteobacteria*	*γ-proteobacteria*	Total
**Phosphate**	solubilizers	15	4	90	6	9	124
	non-solubilizers	40	8	182	14	9	253
	total	55	12	272	20	18	377
	% solubilizers	27%	33%	33%	30%	50%	33%
**Ammonium**	producers	19	3	108	9	8	147
	non-producers	36	9	164	11	10	230
	total	55	12	272	20	18	377
	% producers	35%	25%	40%	45%	44%	39%
**IAA**	producers	2	0	13	1	1	17
	non-producers	53	12	259	19	17	360
	total	55	12	272	20	18	377
	% producers	4%	0%	5%	5%	6%	5%
**CMC**	degraders	9	0	62	4	7	82
	non-degraders	46	12	210	16	11	295
	total	55	12	272	20	18	377
	% solubilizers	16%	0%	23%	20%	39%	22%
**Siderophore**	producers	17	2	114	5	6	144
	non-producers	38	10	158	15	12	233
	total	55	12	272	20	18	377
	% producers	31%	17%	42%	25%	33%	38%

IAA, indole acetic acid; CMC, carboxy-methyl cellulose.

The direct and indirect PGP abilities were displayed by strains belonging to different classes (Table 1): a relative high percentage of strains within Alphaproteobacteria, Bacilli and Gammaproteobacteria classes are able to solubilize phosphate (33%, 33% and 50%, respectively); all classes showed ammonia producer strains (ranging from 25% within Alphaproteobacteria to 45% within Betaproteobacteria); among Gammaproteobacteria the highest percentage of lytic enzyme producers (39%) were found while the largest number (114 out of 272, 42%) of siderophore producers were recorded among Bacilli. Strains resulting positive to IAA and IAA-like molecules were distributed inside Bacilli (13/252), Actinobacteria (2/55), Betaproteobacteria (1/20) and Gammaproteobacteria (1/18). No strain belonging to Alphaproteobacteria was found able to synthetize IAA and IAA-like molecules or lytic enzymes. 26% of total strains did not have any of the PGP traits tested (S1 Table). Numerous strains had more than one of the checked capabilities (mainly distributed among Bacilli but also present among Actinobacteria and Gammaproteobacteria, Table 1): 88% shared two, 29% three and 2% four PGP abilities, respectively; strain 83, namely *Pantoea agglomerans*, and strain 287, namely *Bacillus* sp., solubilized phosphate, produced ammonium and lytic enzymes, secreted siderophores and synthesized IAA and IAA-like molecules.

### PGP strains change *Arabidopsis* root architecture

**Fig 1:**
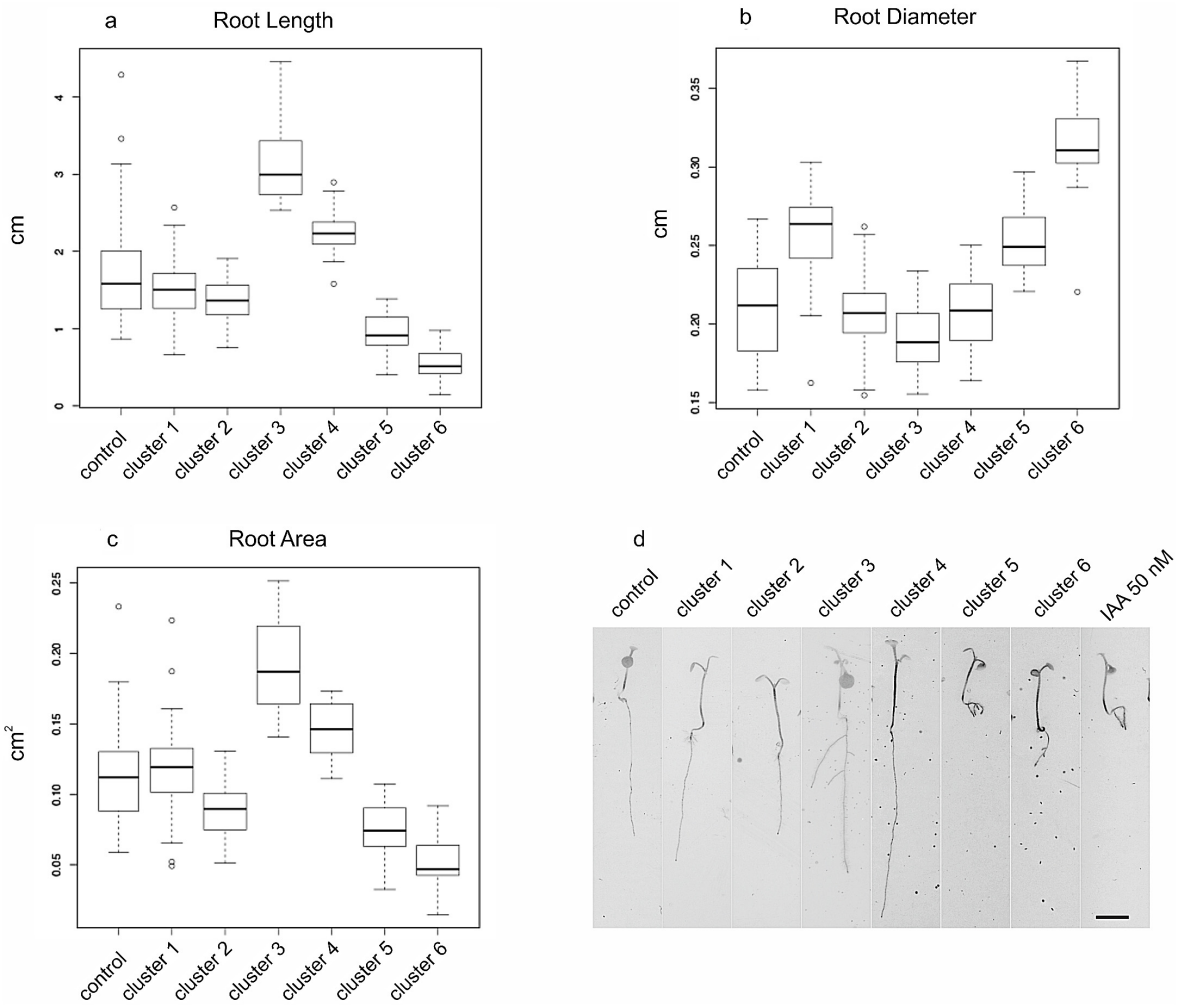
Effects of bacterial strains on *Arabidopsis* root architecture. a-c) supervised model based clustering performed on the variables a) length b) diameter and c) area of the roots. d) *Arabidopsis* plantlet phenotypes (after two weeks of growing) grouped in different clusters obtained from “model based cluster” analysis in comparison with non-inoculated (control) and 50nM IAA-treated seedlings. Scale bars: 1 cm.

To evaluate in a reliable and reproducible way the effects on root morphology, all the isolated endophyte plant growth-promoting strains were tested on the *A*. *thaliana* ecotype Columbia (Col-0). Seedlings were grown in presence of one bacterial strain for two weeks. S1 Fig provides evidences that the measured parameters were not affected by the number of seedlings per plate. 109 strains out of 377 had a negative effect on germination: none of the 13 seeds/squared plate planted in ½MS medium germinated (see in S1 Table, mclust column, which strains induced no seed germination). The effects of the other 268 strains on the root architecture were analyzed using the dataset obtained with WinRhizo^®^ software and the calculated average values of length, area and diameter of roots were used to group the bacterial effects using a model based Cluster Analysis [32]. This method identifies the optimal number of groups characterized by homogeneous values of area, diameter and length root morphology. As reported in Fig 1, the 268 endophyte strains were divided into the six clusters, corresponding to six classes of response induced in *A*. *thaliana* roots, that were compared with the control cluster (non-inoculated plants).

**Fig 2:**
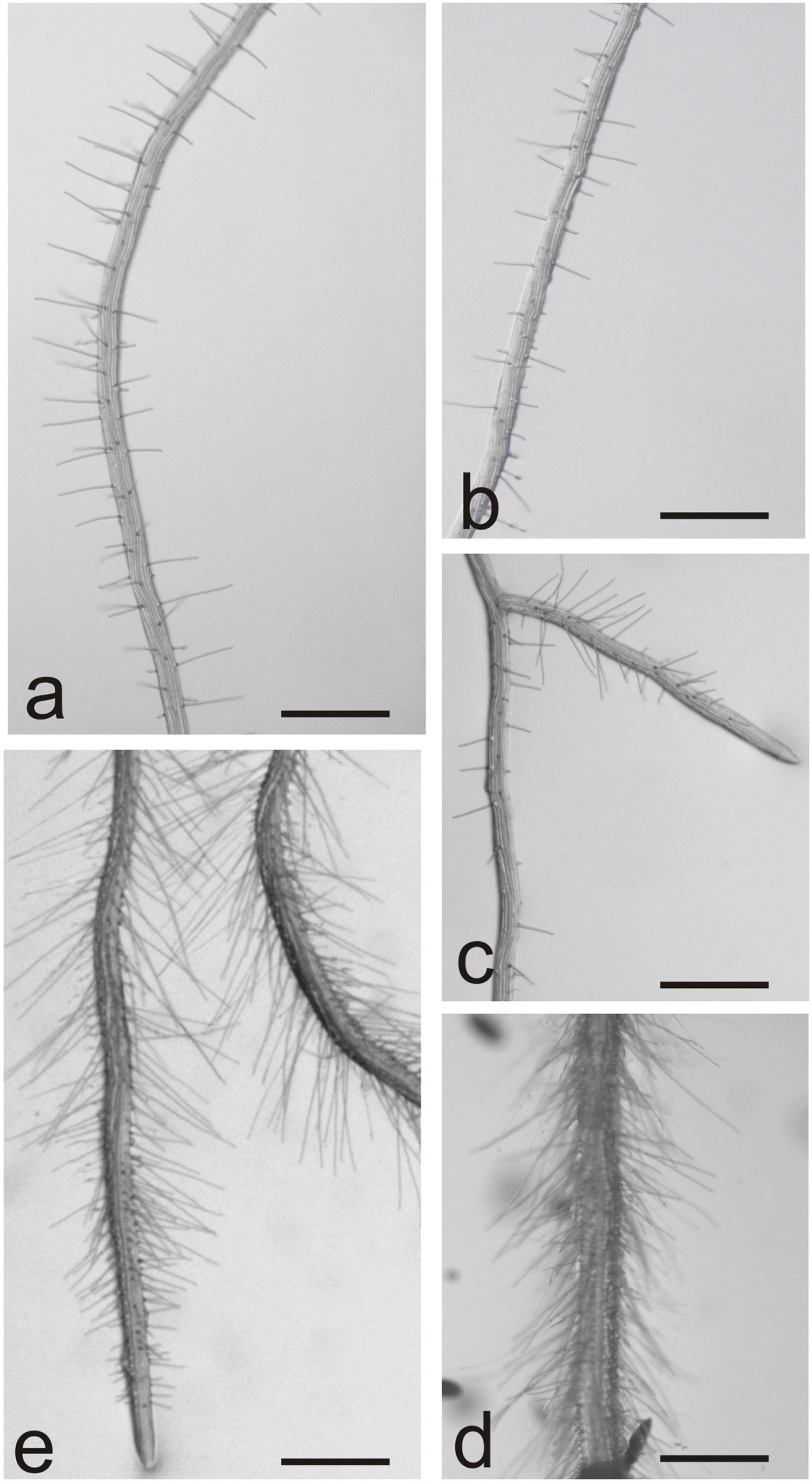
Representative *Arabidopsis* root hairs distribution. The morphology on maturation zone is compared among the six clusters to a) non-inoculated roots and e) 50 nM IAA-treated seedlings. b) seedling roots grouped in cluster 1 and 2, c) seedling roots grouped in cluster 3–4; d) seedling roots grouped in clusters 5 and 6. Scale bars: 500 μm.

Each endophyte cluster is associated with a different level of alteration of the primary root defined as length, area or diameter. Except for the comparison of cluster 1 vs cluster 2 in terms of length (which was not significant P = 0.43) all the other comparisons resulted to be significant (adjusted p-value < 0.05) (S2 Fig). More specifically, clusters 5 and 6 induced a strong shortening of the root, while clusters 1 and 2 were characterized by intermediate root lengths and the other two (3 and 4) induced formation of longer roots compared to the mock (Fig 1a). Although differences in diameter of roots are slight, the dataset analysis revealed differential responses of plant roots (Fig 1b). Clusters 2, 3 and 4 did not show a significant different diameter with respect to the non-inoculated seedlings, while clusters 1, 5 and 6 are characterized by a significant different diameter (p<0.05) compared to untreated seedlings (S2 Fig). Strains belonging to clusters 5 and 6 determined thicker and shorter roots compared to changes induced by all other clusters of strains. Those triggered less marked but significant differences: clusters 3 and 4 caused a reduced root thickness and clusters 1 and 2 effects resulted in a range of intermediate values of root diameters (Fig 1b). In Fig 1c it can be observed that the same differences among the clusters are confirmed in terms of root surface extension effect. Except for cluster 1, all other clusters showed a significant difference compared to non-inoculated seedlings (S2 Fig). One representative *Arabidopsis* seedling for each cluster obtained by the mclust analysis is shown in Fig 1d and an overview of the root hair distribution on root maturation zone, corresponding to completely differentiated primary structure, is presented in Fig 2 for the six clusters compared to non-inoculated and 50 nM IAA-treated seedlings.

Seedling roots included in cluster 1 and 2 showed root length and root hairs distribution and morphology (Figs 1d and 2b) comparable to the non-inoculated plantlets (Figs 1d and 2a). A slight increase in the length of root hairs was observed in clusters 3–4 in which plantlets frequently displayed longer and more branched roots (Figs 1d and 2c); the most dramatic change is evident in plantlets included in clusters 5 and 6: these seedlings developed shorter, thicker and branched root apparati; root hairs appeared longer and denser (Figs 1d and 2d); in this case root length and diameter, density and morphology of root hairs could be compared to 50 nM IAA-treated seedlings (Figs 1d and 2e). Strains belonging to clusters 5 and 6 induced an important shortening of seedling roots that, however, could be compensated by root branching and increased density and/or root hair length leading to a major extension of the exchange surface between root and soil. This is evident also from the biomass measurements at the endpoint of the experiment: no important differences of the total fresh weight were recorded among plantlets belonging to different clusters in respect to the untreated or 50 nM-IAA treated seedlings (not shown). Each of the six classes of root induced-responses in *A*. *thaliana* is triggered by strains that harbor one or more different PGP traits. For instance, cluster 6 encompasses strains able to solubilize phosphate (67%), to produce NH_3_ (56%), IAA and IAA-like molecules (11%) and siderophores (44%), to degrade CMC (11%). All these strains induced development of short, thick, branched roots showing an increase in root hair number and length. A high percentage of the phosphate-solubilizing, ammonia- and IAA-producing strains are included in cluster six but their presence also in other classes of response has to be underlined. Siderophore producing strains are more represented within cluster 4 while CMC degraders are homogeneously distributed in clusters 1–4. A complete overview about the distribution, within each of the six “root response” clusters, of isolated strains that display each of the five tested plant growth-promoting traits is provided in Table 2.

**Table 2:** Percentage of strains, for each of the six clusters of recorded plant root response types, that display the tested PGP traits.

	Mclust					
PGP traits	1	2	3	4	5	6
**Phosphate solubilizers**	11/39 (28%)	17/70 (24%)	18/35 (51%)	19/47 (40%)	29/68 (43%)	6/9 (67%)
**Ammonium producers**	20/39 (51%)	22/70 (31%)	18/35 (51%)	20/47 (43%)	24/68 (35%)	5/9 (56%)
**IAA producers**	3/39 (8%)	3/70 (4%)	2/35 (6%)	2/47 (4%)	2/68 (3%)	1/9 (11%)
**Siderophore producers**	19/39 (49%)	29/70 (41%)	12/35 (34%)	28/47 (60%)	29/68 (43%)	4/9 (44%)
**CMC degraders**	13/39 (33%)	22/70 (31%)	11/35 (34%)	15/47 (32%)	12/68 (18%)	1/9 (11%)

### IAA producing bacterial strains modify the IAA distribution in *Arabidopsis* root

**Fig 3:**
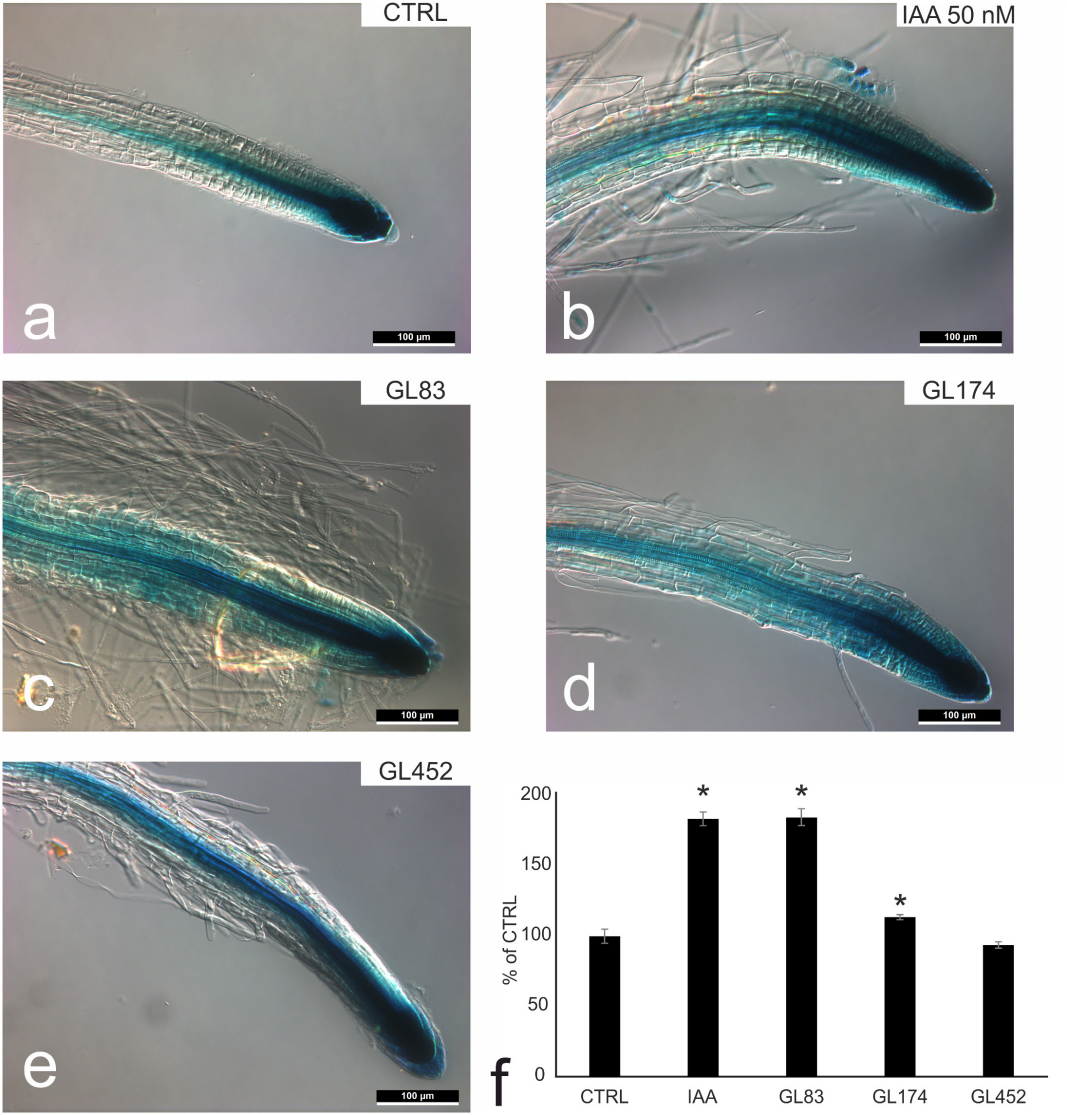
IAA producing bacterial strain effects on *Arabidopsis* roots. a-e) histochemical analysis of 8 day-after-inoculation (dai) *Arabidopsis* DR5::GUS reporter plantlets. A strong induction of β-glucuronidase activity (blue staining) is appreciable in all treated samples (c-e) and in the IAA-treated seedlings (IAA 50 nM, b) compared to non-inoculated seedlings (a). f) 4MU-fluorimetric assay on total protein extract of 8-dai *Arabidopsis* seedlings highlighted a strong induction of β-glucuronidase activity both in the whole IAA and GL83 treated-plantlets. Data were normalized on non-treated seedlings (CTRL) and reported as percentage of the CTRL. Statistically significant at *, P< 0.01.

The previous experiment did not allow to predict which strains could act through IAA production since the 17 IAA positive strains did not group together in the same cluster. They induced different effects on root architecture probably due to differences of IAA and IAA-like molecules production and concentrations in the imposed seedling growth conditions and/or to multiple molecular interactions in which IAA is not the only effector. The 17 positive strains for IAA biosynthesis were further tested to verify their effects on plant development and on the distribution of auxin in the root. *Arabidopsis* seeds harboring the DR5::GUS reporter were sown on the growth medium and seedlings were inoculated with 10^6^ cells per milliliter of one strain per plate. After five days of co-cultivation, seedlings grown in the endophyte-enriched medium showed an altered primary root, mainly shorter compared to the untreated seedlings. This effect was induced by all the 17 tested endophytes. Fig 3 shows representative seedlings grown either in mock conditions (Fig 3a) or with 50 nM IAA (Fig 3b) or in the presence of *Pantoea agglomerans* (Fig 3c, GL83), or *Bacillus licheniformis* (Fig 3d, GL174), or *Bacillus* sp. (Fig 3e, GL452).

The four treatments caused different level of primary root change, and a strong induction of root hairs, a typical effect due to a high concentration of auxin. β-Glucuronidase activity of the *Arabidopsis* reporter lines was analyzed both by histochemical and by quantitative fluorimetric assay. In Fig 3a, a typical auxin distribution within *Arabidopsis* root was observed in mock inoculation: endogenous auxin accumulates in the quiescent center and in columella cells in the root apex. 50 nM IAA treatment (Fig 3b) induced a wider distribution of increased intensity GUS staining in the root meristematic cells. Moreover, a strong increase in the number of root hairs can be observed. Seedlings grown in presence of *Bacillus* sp. GL452, showed a glucuronidase activity similar to auxin treated samples, while the treatment with *Pantoea agglomerans*, GL83, and *Bacillus licheniformis*, GL174, showed a stronger induction of the β-Glucuronidase activity and further increased root hair number. The same samples were also analyzed by quantitative fluorimetric assay and reported in Fig 3f. 10 μg of total protein extracted from entire *Arabidopsis* seedlings were incubated in the presence of 4-Methylumbelliferone-glucuronide and the fluorescent product was measured. β-Glucuronidase activity of the whole plant resulted strongly upregulated in the samples treated with exogenous IAA and with *Pantoea agglomerans*, GL83. A lower but still statistically significant increase was observed in the sample treated with *Bacillus licheniformis*, GL174 while fluorescence yielded in the sample treated with *Bacillus* sp., GL452, resulted comparable to untreated seedlings. These results confirm a IAA biosynthetic activity in the endophytes tested, which could also affect the hormonal homeostasis of the entire plants for two of the strains.

### Selection of candidate PGP strains for future *in vitro*, *in planta* and in field tests

The screening for an array of conventional bacterial PGP abilities *in vitro*, the analysis of bacterial- induced effects on root growth promotion during germination of *Arabidopsis* seedlings and results of *Arabidopsis* seedling inoculation with the auxin-producing strains provided useful indications for a choice of a limited number of promising strains from the Glera established collection, which encompassed 377 strains (Table 3). Two of the strains, namely *Pantoea agglomerans* GL83 and *Bacillus* sp. GL287, had all the tested PGP properties and displayed very diverse effects on *Arabidopsis* root architecture. From the cluster analysis, they were included in two different clusters: *P*. *agglomerans* GL83 induced thick and long roots (cluster 1) and *B*. *licheniformis* GL174 shaped short, thick, branched and hair-rich roots (cluster 5). Three auxin (or auxin like-molecule)-producing strains displayed one additional ability: *Agrococcus baldri* GL13, *Paenibacillus* sp. GL24 and *Bacillus licheniformis* GL174 were able to produce siderophores, lytic enzymes and ammonium, respectively and triggered three different morphological responses of *Arabidopsis* inoculated roots. *Micrococcus* sp. GL74, *Microbacterium flavum* GL89 had only one among the tested PGP abilities and they were included in two bacterial clusters inducing strong modifications to root architecture. Two strains, belonging to Bacilli, showed two out of five tested PGP activities: *Bacillus* sp. GL169 and *Bacillus herbersteinensis* GL 186 solubilized phosphate and ammonium; *Micrococcus* sp. GL168, and two Bacilli (GL412 and GL452) mobilized phosphate and ammonium from substrates and secreted lytic enzymes or siderophores, respectively.

**Table 3:** Assayed PGP traits of selected strain, their cluster attribution and their taxonomical identification.

	Plant growth-promoting traits						
Strains	Phosphate solubilization	Ammonium production	IAA production	Siderophore production	CMC degradation	Cluster PGP analysis	Taxonomy
GL13	-	-	+	+	-	1	*Agrococcus baldri*
GL24	-	-	+	-	+	2	*Paenibacillus* sp.
GL74	-	+	-	-	-	2	*Micrococcus* sp.
GL83	+	+	+	+	+	1	*Pantoea agglomerans*
GL89	-	-	-	-	+	5	*Microbacterium flavum*
GL168	+	+	-	-	+	3	*Micrococcus* sp.
GL169	+	+	-	-	-	3	*Bacillus* sp.
GL174	-	+	+	-	-	3	*Bacillus licheniformis*
GL186	+	+	-	-	-	2	*Bacillus herbersteinensis*
GL287	+	+	+	+	+	5	*Bacillus* sp.
GL412	+	+	-	+	-	2	*Bacillus* sp.
GL452	+	+	-	+	-	5	*Bacillus* sp.

## Discussion

The work hereby presented is part of a wider project that aims to characterize and select endophytic bacterial strains that could be potentially used as biofertilizer and biocontrol agents in vineyards. After a thorough surface sterilization of explants, 377 strains were isolated from inner vegetative tissues of *V*. *vinifera* cv. Glera. A molecular characterization using the ARDRA technique [35], indicated that the community, limited to cultivable fraction, displayed a high biodiversity [24].

In this work, we analyzed some of the activities of culturable bacterial strains directly and indirectly involved in plant growth promotion. As reported [20, 36] endophytic bacteria promote plant growth improving mineral nutrition of the plant largely by solubilization of inorganic phosphate, production of ammonia, secretion of siderophores and production of IAA and IAA-like molecules. In this study we demonstrated that 124 out of 377 assayed strains were able to solubilize phosphate, increasing its bioavailability to plant tissues. Although present in the soil, 95% of phosphorus is not available for absorption due to insoluble salts. The production of organic acids (acetate, succinate, citrate, gluconate) by bacteria induces acidification of the soil and provides the release of phosphate ions from inorganic aggregates. In addition, bacteria can express phytases and phosphatases, which make phosphate ions available from organic compounds [18]. The assessment of the production of ammonia was qualitative and 39% of culturable endophytic bacterial strains, isolated from Glera tissues resulted positive. Nitrogen availability is the main limiting factor in crop productivity and many endophytes developed the ability to produce ammonium or to convert nitrates (NO_3_^-^) to nitrites (NO_2_^-^) in anoxic conditions. In addition, bacteria derive energy from this conversion that may be useful for the metabolism of the host in a final analysis [8]. Production of ammonium is a connotative capacity attributed to soil bacteria as well as PGPR/PGPB [37], but it was also found in endophytes, known to spend part of their life cycle in the soil [20]. Both these two PGP activities, phosphate solubilization and ammonia production, have been reported also among the array of PGP abilities of strains isolated from cultivar Barbera plant tissues [38] and from grapevines influenced by environmental factors as they were cultivated in three different Mediterranean regions [39]. They are common PGP traits that maintain a great basic functional PGP potential in endosphere of grapevine root and stem tissues.

The production of siderophores, molecules able to bind iron leading to formation of iron-siderophore complexes that bacteria and plants can easily internalize and metabolize, is a widely diffuse PGP ability that also can exert a biocontrol role limiting fungal pathogen germination [9]. Glera tissues host a great number of siderophore producers (38% of the total strains), distributed among different classes but mainly present within Bacilli. This widespread direct PGP and indirect biocontrol ability [9, 8, 40] has often been detected for culturable microbiome in grapevine [38, 39, 41]. During the plant colonization process, bacteria are helped by the secretion of cell wall degrading enzymes that permit bacteria to loose cell walls penetrating in the roots helping movements within tissues [36, 42]. The secretion of these lytic enzymes by Glera isolated endophytes, detected in 22% of the total bacterial population, make them prone to interact with the host plant and translocate within the plant-helping niches establishment. This facilitation of penetration has already been reported in grapevine that can be colonized by *Burkholderia phytofirmans* strain PsJN spreading throughout organs and tissues [43, 44].

It is known that grapevine tissues harbor endophytic communities that have been identified both by cultivation-dependent and independent methods in diverse cultivars [38, 39, 45–47]. In grapevine many researches are focused on bacterial abilities to protect the plant against pathogen-induced diseases [48–52]; less indication is present in specific literature about direct effects of bacterial plant growth-promoting traits. Many conventional PGP traits displayed by the endophyte collection, considered in this work, have already been demonstrated to be involved in enhancement of chilling resistance of grapevine plantlets [53], to improve grapevine adaptation and survival to induced-water stress [38]. Moreover this PGP potential is widely present in bacterial communities colonizing vineyard located in diverse environments [39].

Within bacteria isolated from surface-sterilized Glera tissues, many displayed more than one PGP trait and two of them resulted positive to all the tested direct and indirect activities. Strains sharing multiple PGP abilities are indeed the most interesting for plant growth promotion, from a nutritional point of view; they could have an important influence on plant physiology and consequently on morphology and root architecture that has a crucial role in acquiring nutrients and water, which directly affects the growth of above-ground plant organs [9, 15]. Several evidences have demonstrated that PGPEs induced modifications of roots that enhance soil exploration, i.e elongation of the primary root, increase of the number of lateral roots, root hairs, leading to the increase of the total root surface [14]. All Glera endophytes were assayed on the model plant *A*. *thaliana* to have an overview of their effects on root morphology. This plant species, already used for this purpose [54] provides homogeneous responses in short time allowing a reliable analysis of the effects. Data derived from numeric parameters representative of root length, average diameter and total root surface, statistically analyzed, allowed to group bacteria according to different effects on root morphology compared with non-inoculated plantlets; some strains caused an elongation of the root and an increased root surface and others induced enlargement of the average diameter and triggered a reduction in length but also a rise of the number and length of root hairs that sharply contribute to an extension of root exchange surface. In addition to the influence on mineral plant nutrition and water uptake, it is well demonstrated that PGBE play an important role in plant growth promotion by production of auxins an IAA-like molecules, via an alternate tryptophan-dependent pathway, through indolepyruvic acid [9, 29]. Using knock-out mutants for the pathway of auxin biosynthesis it has been demonstrated that IAA produced by *Azospirillum brasilense* increased the root length and surface in wheat plants inoculated with the strain [55]. Moreover this auxin-producing bacterium induced differential gene expression in *Arabidopsis* roots after inoculation with either wild-type or an auxin biosynthesis mutant [54]. This PGP activity is one of the most common spanning among bacteria isolated by numerous plant species [9] and it has also been reported for strains isolated from grapevines grown in different regions of Italy and of the Mediterranean area [38, 39]. In this work 17 strains out of 377 isolates were able to synthetize IAA and IAA-like molecules *in vitro* in the presence of tryptophan. This small recorded percentage of *in vitro* IAA producers, tested on the *Arabidopsis* root system, was not grouped together in an only cluster but they were assigned to different clusters representative of diverse morphology of plant roots. It is known that a low concentration of auxin causes an elongation of the root whereas a high concentration reduces root length, increases root diameter, triggers lateral root emergence and root hair density [9, 29, 55, 56]. From the analysis of the distribution of strains, harboring one or more plant-growth promoting traits, in each cluster of *Arabidopsis* “root responses” it is therefore clear that observed root modifications were the result of multiple interactions in which IAA plays an important role but is not the only effector. Recent literature has reported the importance of microorganism action to increase the phosphorus availability that in turn altered root traits: enhancement of lateral root number, greater root biomass and longer, denser root hairs. Moreover an influence of phosphate solubilization on auxin-dependent lateral root formation has been underlined [57, 58]. A clear involvement of bacterial synthetized IAA and IAA-like molecules was confirmed by using *Arabidopsis* seedlings, harboring the auxin responsive marker DR5::GUS. The influence of bacterial IAA-producers led to a sharp increase on plant endogenous auxin levels after inoculation. The IAA distribution, in treated plant roots, was appreciable even in the cortical layers and sometimes in the root epidermis in addition to the root meristematic and columella cells that showed an important reporter activity also in non-inoculated plantlets. These observations support a direct involvement of IAA produced by bacteria in determining modifications in root architecture and an increase in root hair number. On the other hand, the effect of the other bacterial activities on root morphology needs to be investigated [59] as this result could depend on differences in IAA production rate, on growth conditions, or on the presence of multiple overlapping molecular mechanisms, which differentiate the response of the plant [15, 60].

In fact, it is widely accepted that endophytes could directly impact plant growth by altering the endogenous levels of auxin, cytokinins, gibberellins, ethylene and other plant hormones [14, 56]. However, it has been proposed that endophytes could adopt an additional wide variety of strategies, which may include the production of compounds capable of interfering with the synthesis, degradation, transport or signaling of phytohormones [15, 61, 62].

At the end of our analysis, based on biochemical assays, on the analysis of *Arabidopsis* development and on IAA involvement, we were able to assess the overall effect on growth and discern the most promising candidates as potential growth promoters. Among the chosen strains seven belong to the Bacilli class. *Bacillus* was the most abundant and represented genus in Glera grapevine endophyte community and has been frequently reported in other grapevine cultivars both by culture-dependent and independent methods of isolation [39, 45, 47]. This result reflects the ubiquitous distribution of this genus, which colonizes many cultivated plant species and has already been employed in agricultural field inoculants. Several species of *Bacillus* are known for their ability to produce a broad spectrum of molecules with biological activities useful in several applications [63–65]. Also the *Pantoea* genus has been often described within the grapevine microbiome and it has been demonstrated to mediate grapevine resistance against *Botrytis cinerea* [49, 50]. The use of bacterial based formulations can improve the nutritional status of crops without deleterious effects on the ecosystem (i.e. mineral fertilization causes the depletion of the biological component of the soil, also important for the maintenance of its fertility and structure [66]) and also stimulate plant growth in response to specific stress conditions [14, 67].

Thus endophyte plant growth-promoting effects should be now translated and studied in the development of the grapevine complex root architecture. As indicated in literature [68] the actual growth-promoting activity of beneficial bacteria is anyhow subjected to multiple complex events involving both plant and bacteria and requiring their efficient compatibility as well as compliance with the remaining environmental biota and conditions. While tests on model hosts, i.e. *Arabidopsis*, allow easier screening in a genetically and microbiologically controlled setting, the ultimate trials for perspective PGP inoculants have to be conducted *in vivo* and eventually in field conditions on the intended crop, which will constitute future developments of the present line of research.

## Supporting Information

S1 Fig Representative *Arabidopsis* root length of 13 plantlets at final time point of growth. Two week old plant roots do not interact or touch each other: mock seedlings (a); 50nM IAA-treated seedlings (d); seedlings belonging to clusters 1 and 2 (b), to clusters 3 and 4 (c); to clusters 5 and 6 (e).
(TIF)

S2 Fig Pairwise tests comparing the mean of area, diameter and length variables across clusters. Specifically Tukey test has been used to take into account the multiple testing problem. For each variable the contrast (the clusters compared), the difference of the clusters means, and the adjusted p-values are reported.
(TIF)

S1 Table Overview of PGP tested activities, cluster attribution and taxonomic identification of all isolated strains. (XLSX)
