# Supplementary figures and images for: Beneficial Bacteria Isolated from Grapevine Inner Tissues Shape Arabidopsis thaliana Roots
Source: PLoS One. 2015 Oct 16;10(10):e0140252. doi: 10.1371/journal.pone.0140252 (PMC4652591; doi:10.1371/journal.pone.0140252)

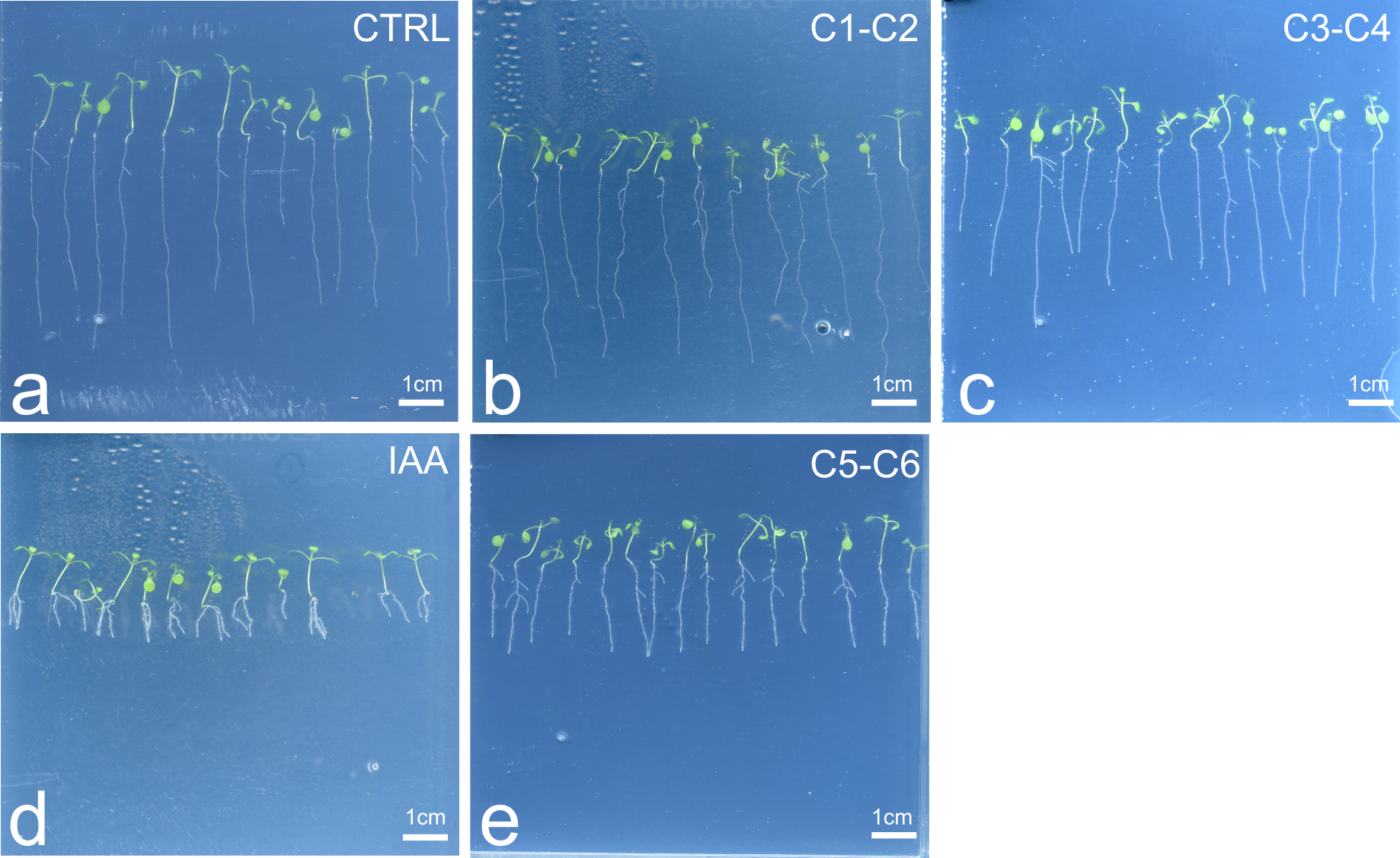

Supplement: S1 Fig — Two week old plant roots do not interact or touch each other: mock seedlings (a); 50nM IAA-treated seedlings (d); seedlings belonging to clusters 1 and 2 (b), to clusters 3 and 4 (c); to clusters 5 and 6 (e). (TIF) [file pone.0140252.s001.tif]

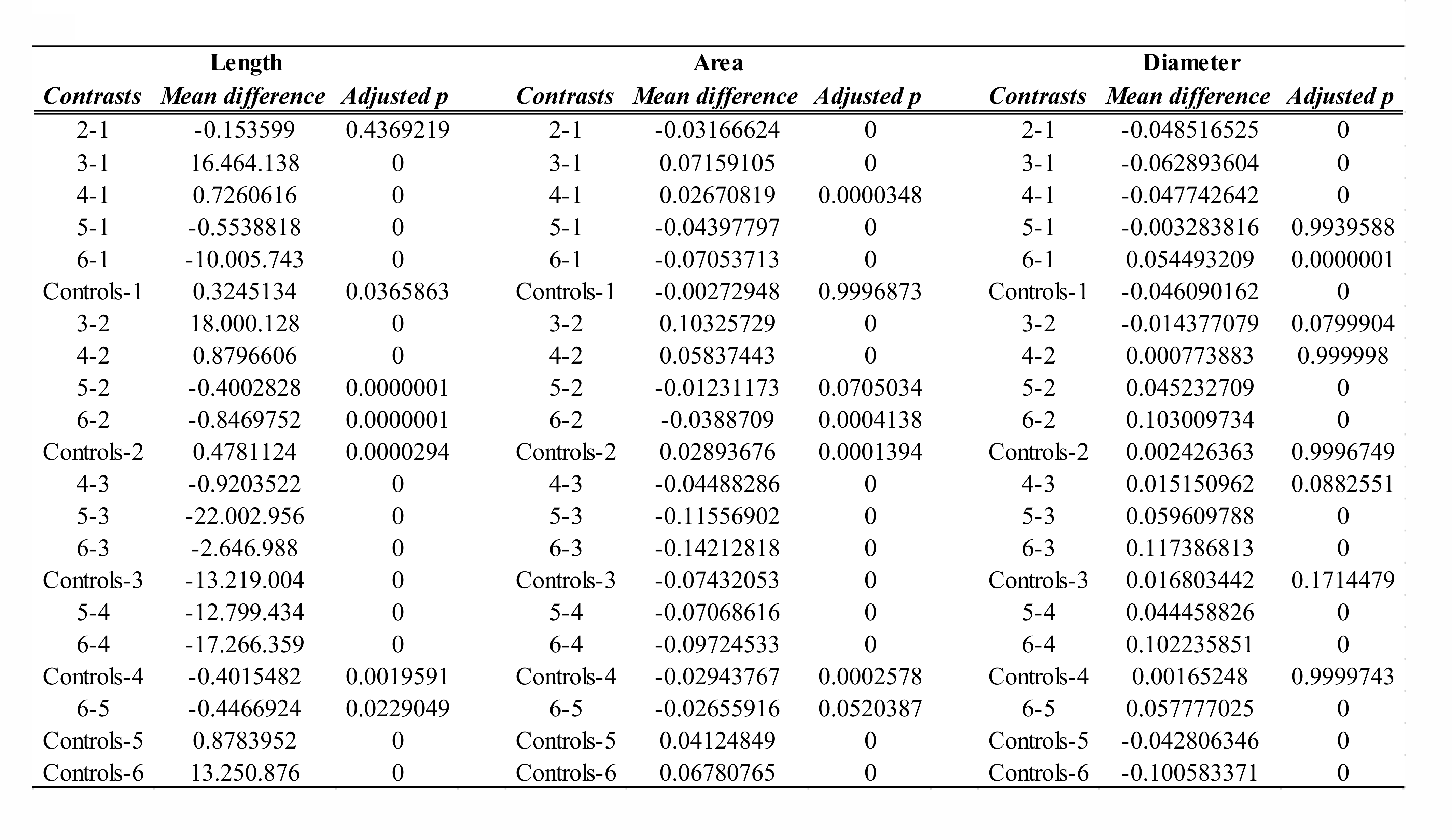

Supplement: S2 Fig — Specifically Tukey test has been used to take into account the multiple testing problem. For each variable the contrast (the clusters compared), the difference of the clusters means, and the adjusted p-values are reported. (TIF) [file pone.0140252.s002.tif]
